# 

**Published:** 1860-09

**Authors:** 


### RECEIPTS FOR THE JOURNAL, FOR JULY & AUGUST, 1860

| | | | |
|---|---|---|---|
| J S & Z H Whitmire, Ill,... | Vol, | 3 | 2 00 |
| E Evans, Ill. | " | 3 | 2 00 |
| J K Foster, Iowa, | " | 3 | 2 00 |
| S L Urmston, Ark., | " | 3 | 2 00 |
| E Vogler, Ill | " | 2–3 | 4 00 |
| Amos P Finch, Ill | " | 3 | 2 00 |
| Wm McKnight, Ill...last ½ | " | 3 | 1 00 |
| F K Bailey, Ill | " | 1 | 2 00 |
| J B W Lewis, Mich | " | 1–2 | 4 00 |
| A P Adams, Wis | " | 3 | 2 00 |
| A J Larimore, Ind | " | 3 | 2 00 |
| C J Taggart, Wis | " | 3 | 2 00 |
| J B Thurman, Ill ..½ | " | 3 | 1 00 |
| H C Martin, Iowa | " | 3 | 2 00 |
| L W Lovell, Mich | " | 3 | 2 00 |
| James A Roman, Min | " | 3 | 2 00 |
| Jno Cleoves, Iowa, ½ vol 3 ½ | " | 4 | 2 00 |
| E A Rogers, Ill | " | 1–2–3 | 6 00 |
| J Blount, Ill | " | 3 | 2 00 |
| N P Holden, Ill | " | 2 | 2 00 |
| A Wood, Ind ¼ | " | 3 | 50 |
| G C Holmes, Ill ½ | " | 3–1–2 | 5 00 |

### TO SUBSCRIBERS IN ARREARS.

Many readers of the *Journal* are indebted to us on accounts of one, two, and three years standing. We have been extremely lenient in the matter of collections, knowing the difficulty of obtaining money that many have experienced during the recent period of financial depression; but the abundant harvest of the present season, throughout the entire Northwest, does away with all apology for a continuation of such remissness. Therefore, in the September number, we shall inclose to each indebted subscriber his bill, and we trust, that with this reminder, all will come promptly forward to pay us what is justly our due.

